# Differences in Motor Development between Preterm Infants and Full-Term Preschool Children

**DOI:** 10.3390/children11020252

**Published:** 2024-02-16

**Authors:** Alicia Cuesta-Gómez, Pilar Fernández-González, María Carratalá-Tejada, Inmaculada Aguilar-Bejines

**Affiliations:** 1 Motion Analysis, Ergonomics, Biomechanics and Motor Control Laboratory (LAMBECOM), Department of Physical Therapy, Occupational Therapy, Rehabilitation and Physical Medicine, Faculty of Health Sciences, Rey Juan Carlos University, 28922 Madrid, Spain; alicia.cuesta@urjc.es (A.C.-G.); maria.carratala@urjc.es (M.C.-T.); 2 Crecer con mi Fisio|Fisioterapia Infantil, CL Estrasburgo, 41012 Sevilla, Spain; inmaguilarfisioterapia@gmail.com

**Keywords:** premature birth, preschool child, motor skills, educational early intervention

## Abstract

Although advances in obstetric and neonatal care have improved the survival of preterm infants, many studies document the increased risk of motor and sensory neurodevelopmental abnormalities that can hinder school progress. The aim of this study was to analyze the differences in gross and fine motor development in children born preterm and full term aged 3 to 6 years using the Peabody Developmental Motor Scales 2 (PDMS-II). Fifteen preterm and fifteen term children, matched for age and sex, participated in this study. They were evaluated with the PDMS-II scale. The scores obtained in the PDMS-II scale showed statistically significant differences (*p* < 0.05) in all subscales except for the “grasping” subscale. No dissimilarities were found between children who attended an early intervention program and those who did not participate, nor was there any correlation between week of gestation and birth weight and motor development in preschool. The results obtained show that differences are found with respect to motor development, with lower scores for those born preterm compared to children born at term. No statistically significant difference was found between preterm children who attended early intervention and those who did not. No correlation was found between motor development at preschool age and birth weight and gestational age.

## 1. Introduction

Although advances in obstetric and neonatal care have improved the survival of preterm infants, many studies document the increased risk of motor and sensory neurodevelopmental abnormalities, such as cognitive dysfunction and behavioral disorders, that can hinder school progress [1,2,3]. Low birth weight and the week of gestation in which infants are born are inversely proportional to the degree of possible alterations they may present and to the time in which differences with full-term children persist [4]. On the other hand, the impact of being born preterm tends to diminish as the child grows older [5].

Alterations in motor development are the most frequent sequelae in premature infants [6]. The male sex, gestational age, persistent ductus arteriosus, grades 3 and 4 intraventricular hemorrhage, periventricular cystic leukomalacia, postnatal corticosteroid administration, bronchopulmonary dysplasia, surgical interventions, and slower intrauterine growth are, among others, predictors of risk of motor impairment [7]. These children tend to reach motor milestones later than full-term children during infancy and childhood [5]. Some studies show significant differences in the motor development of preterm children up to 2 years of age, in relation to children born at term or in relation to their age [8,9].

During the preschool period (3 to 5 years), children begin to develop more complex motor milestones such as improving postural control, increasing walking speed, starting to run, climbing up and down stairs, jumping... They also begin to combine gross and fine motor skills and learn tasks such as throwing and catching balls [10]. And it is at this age that the diagnosis of milder motor problems that can lead to limitations in motor control, affecting children’s levels of interaction with and participation in society, is carried out [11]. Therefore, long-term follow-up and tailored intervention are considered important to optimize the development of premature infants [12,13].

Minor neurological dysfunctions (MNDs) is a term used to describe “neurological alterations in children who do not develop Cerebral Palsy, but have some neuro-motor alteration”, such as difficulty in posture, regulation of muscle tone, balance, coordination, or presence of abnormal reflexes [14]. MNDs have a high incidence in premature children, often still present at 5 years of age, and can have educational repercussions [14,15,16].

An increased likelihood of developing developmental coordination disorders (DCDs) [17], defined as “motor problems that interfere with academic performance or activities of daily living and cannot be explained by medical, neurological or cognitive deficits” [18], has also been documented in preterm or low-birth-weight school-age children. And, once again, it has been shown that they have a relationship with cognitive performance [19].

Evensen et al. [20] in their 2020 study reviewed the long-term motor outcomes in preterm or low-birth-weight children without neurological damage. They described a wide variety of findings: very preterm or low-birth-weight children scored lower on developmental scales than full-term children at ages up to adolescence [21]; developmental coordination disorders were more frequent in very preterm or low-birth-weight children; developmental impairments or delays may persist at different ages of follow-up. The prevalence of motor disturbances was significantly higher than in controls (children born at term), although there were few data indicating that these disturbances persisted into adulthood and this, they believe, may be due in part to the lack of validated assessment tools for young people [20]. Motor dysfunctions were related to psychiatric problems, as well as inattention and symptoms of anxiety and depression in adults. This can have negative effects on quality of life, academic achievement, and involvement with the environment [20].

To our knowledge, most of the studies that exist in the current literature focus on very premature or low-birth-weight infants and children at early ages, most of them between 0 and 2 years of age. It must be taken into account that premature infants may carry risks in their long-term development [17,22].

For this reason, it is important to know whether differences and alterations in motor development continue to exist at preschool ages in order to be able to detect possible warning signs in time to provide early and individualized intervention for each case and try to reduce future consequences. This study hypothesizes that preterm infants exhibit significantly different motor development trajectories compared to full-term preschool children.

The aim of the present study was to analyze the differences in gross and fine motor development in children aged 3 to 6 years born preterm and those born at term using the Peabody Developmental Motor Scales 2 (PDMS-II). 

The secondary objectives were to compare the motor development of preterm children included in an early care program with those who were not, and to estimate the relationship between PDMS-II scores and the birth weight and week of gestation of preterm children.

## 2. Materials and Methods

This was a cross-sectional, analytical, single-blind, case–control, observational study. The sample was recruited by non-probability sampling of consecutive cases.

The present study was conducted following the structure and international recommendations of the STROBE checklist for observational studies.

### 2.1. Ethical Aspects

This study was approved by the Ethics Committee of the Universidad San Pablo-CEU with reference number 653/22/TFM, in accordance with the ethical principles for medical research involving human subjects of the Declaration of Helsinki. 

Written informed consent was obtained from the parents of each participant.

### 2.2. Participants

Voluntary participation was solicited from children in different educational centers in Madrid and Sevilla (Spain). Participants were recruited into the study if they met the following inclusion criteria: children between 3 and 6 years of age born prematurely with the capacity to understand verbal commands and whose parents or legal guardians had agreed to be included in the study and signed an informed consent form. 

The following exclusion criteria were considered: diagnosis or suspicion of some type of pathology, neurological damage, or cognitive, language, or behavioral alterations. 

For the control group, voluntary participation was requested from children born at term between 3 and 6 years of age who did not present a diagnosis or suspicion of any type of pathology, neurological damage, or cognitive, language, or behavioral alterations. The control group was matched by age and sex with the sample of the preterm group.

### 2.3. Procedure

First of all, the search for a sample was carried out. For this purpose, several schools and nursery schools were contacted and three agreed to participate in the project. 

The teachers of the different infant classes selected children who met the selection criteria. Their families were sent an informed consent form, a brief explanatory infographic, a data collection sheet requesting name and surname, sex, date of birth, week of gestation, type of delivery, birth weight, place of birth, number of siblings and place of birth, relevant medical data, hospital admissions, participation in an early care program or in extracurricular activities, and year of schooling, and a letter introducing the principal investigator and the project.

Once the informed consent was signed, the scale was administered by the principal investigator to the children included in this study in the centers themselves during school hours. The children left their classrooms randomly and anonymously, accompanied by their tutors, until they arrived at the evaluation site (psychomotor gymnasium or school playground), where the principal investigator was located without having knowledge of the group to which the child belonged. Adequate material and environmental conditions were provided to guarantee the comfort and privacy of the participants. 

During the assessment, the researcher did not know to which group each child belonged, since the data recording sheets were not collected until the end of the assessment.

### 2.4. Outcome Measures

The main variable to be studied was the “degree of motor development”, measured using the PDMS-II [23]. This is a scale that measures gross and fine motor development in children. It is used to determine whether a child’s motor development is in accordance with his or her age, as well as being a predictive and validated tool for research. 

It consists of 241 items divided into 6 subscales; all items reflect everyday experiences such as going up or down stairs, throwing or catching a ball, making a tower with cubes, and cutting, among others.

Within gross motor skills we find reflexes (evaluable up to one year of life); static balance (30 items related to static stability, such as standing on tiptoes, standing on one foot...); locomotion (89 items that include jumping in different ways, walking backwards, speed in running...); and object manipulation (24 items related to throwing, kicking, and catching a ball, aiming, or spatial–temporal coordination).

With respect to fine motor skills, there are two sections: grasping (including 26 items such as buttoning and unbuttoning buttons, how to hold a pencil...) and, finally, visual–motor coordination (72 items, including the construction of towers and figures with cubes, imitation of strokes and geometric figures, cutting, threading a cord...).

The age (in months) of the child dictates the item with which the test begins. Each of the items is scored 0, 1, or 2, according to the scoring criteria we find written on each item. The lower level is established when the child receives a score of 2 on three consecutive items; all items below the lower level are scored with 2. The upper level or ceiling is established when the child scores 0 on three consecutive items; after this, the test is suspended. The raw scores obtained in each subsection should be converted into standard scores and percentiles for comparison. In addition, by adding the standard scores of each subsection, the gross motor quotient (GMQ) and fine motor quotient (FMQ) are obtained, and the total motor quotient (TMQ) can be obtained.

This scale has excellent psychometric properties. Griffiths et al. [24] conducted a systematic review of the psychometric properties of different tools that assess motor development in children. In their review, they included three studies which evaluated the psychometric properties of the PMDS-II: Hua et al. [25] described the scale’s internal consistency as excellent, with good reliability; both content and criterion validity were excellent. Wuang et al. [26] assessed the psychometric properties of the scale for children with intellectual difficulties. They found fair internal consistency and sensitivity and good reliability, criterion validity, and measurement error. Finally, the scale’s own authors, Folio et al. [23], described good internal consistency, structural validity, and hypothesis testing; poor measurement error and criterion validity; and excellent content validity. 

In addition, in 2014, Tavasoli et al. [27] studied the reliability and validity of the scale in very-low-birth-weight preterm infants. For reliability, they studied internal consistency by means of a 0.90–0.92 Cronbach’s alpha and an intraclass correlation coefficient of 0.98; on the other hand, validity was examined by comparing known groups, resulting in a valid tool for the assessment of motor development in this population.

### 2.5. Statistical Analysis

Statistical analysis was performed using the SSPS statistical software system (SSPS Inc., Chicago, IL, USA; version 27.0). Descriptive analysis was performed for all variables. The results were expressed as medians and interquartile ranges. 

The Shapiro–Wilk test was used to test whether the variables followed a normal distribution. The hypothesis that the variables did not have a normal distribution was accepted due to the result of the test and the verification of the histograms of each variable. Therefore, the Mann–Whitney U test, a non-parametric test for unrelated samples, was used to compare the variables in both groups. Spearman’s correlation coefficient was used to correlate the variables. 

The statistical analysis was performed with a confidence level of 95%, so that values with a *p* < 0.05 were considered significant.

## 3. Results

The final sample consisted of 30 subjects (16 males and 14 females), 15 of whom were born prematurely and the other 15 were born at term. The age of the sample ranged from 39 to 69 months; the mean was 47.93 ± 9.89. Within the preterm group, none were extremely preterm (born at fewer than 28 weeks’ gestation). The mean was 32.93 weeks gestation and 1979.66 g at birth (Table 1).

Regarding the analysis of the scores on the PDMS-II scale, statistically significant differences were observed between the two study groups. Preterm infants generally obtained lower scores compared to the group of children born at term. Statistically significant differences were observed in all subscales of the PDMS-II scale, with the exception of the “Grasping” subscale: static balance (*p* = 0.001), locomotion (*p* = 0.001), and object manipulation (*p* < 0.01). In the fine motor subscales, hand–eye coordination (*p* = 0.011) also obtained statistically significant differences. As for the quotients, statistically significant differences were observed in the GMQ (*p* < 0.01), FMQ (*p* = 0.021), and TMQ (*p* < 0.01) (Table 2).

No differences were observed between preterm infants who participated in an early care program and those who did not (Table 3).

With respect to the analysis of the correlations between the different subscales and ratios and the week of gestation and birth weight (Table 4), no statistically significant differences were found between any of the correlations performed.

## 4. Discussion

The main hypothesis of the present study was that, after 3 years of age, there are still differences in motor development between children born prematurely without any type of pathology and children born at term; although the scores obtained on the PDMS-II motor development scale for all the children in the study were within the percentiles, it was observed that the group of children born at term had significantly higher scores than the preterm children. Likewise, it was proposed to estimate the difference between preterm children who had attended an early intervention program and those who had not; no differences were found between the two. Finally, the correlation between the degree of motor development and the gestational weight and age at birth was studied and no differences were found between the correlations studied. 

Prior to our study, there had already been similar investigations in the literature that shared the finding of differences between preterm and full-term preschool children: Kilbride et al. [28] compared the development of 25 preterm preschool children with their siblings; to try to avoid any bias related to the child’s environment, they used the gross motor part of the PDMS-II scale at 3 or 5 years of age, and the results were significantly lower in preterm children in terms of the gross motor quotient (*p* = 0.04). In the present study, significant differences were also found for the gross motor quotient of the same scale (*p* < 0.01), although the environment of the participating children was not taken into account. 

Huddy et al. [2] studied educational impairment in 117 late-preterm children aged 7 years, finding that 12% of them had low scores in physical education. As in our study, lower scores were found in preterm children, even though they were late preterm, and they observed how these differences can sometimes persist until school age.

Oliveira et al. [29] found once again that preterm children without severe sequelae had a higher frequency of developing motor coordination and attention disturbances. They assessed the motor development of 23 children aged 5 and 6 years with the Developmental Coordination Disorder Questionnaire for Families and the Movement Assessment Battery for Children scale (MABC), with preterm children scoring worse (*p* = 0.02). Torrioli et al. [30] conducted a similar study, also using the MABC to measure motor development in children aged 4 to 6 years; 70.6% of the preterm children without neurological damage who participated in the study scored abnormal or borderline. Hoff Esbjørn et al. [31] again reported that the scores obtained by children born prematurely at age 5 years were significantly worse on the MABC scale (*p* < 0.01). Although these three studies use a different motor development scale than the one used in the present study, they coincide in that lower scores were obtained by the group of premature children at ages similar to those in our study, with a statistically significant difference for the total motor quotient (*p* < 0.01).

In our investigation, no significant differences were found in the “grasping” subscale score. To our knowledge, there is no previous research comparing the motor development of preterm and term infants using the PDMS-II and breaking down all the items in order to observe differences or similarities in this area. FitzGerald et al. [32], in addition to motor development using the MABC and developmental coordination disorder with a family questionnaire, measured grip strength in both hands of 123 preterm infants compared to 128 full-term infants using a calibrated digital hand dynamometer. They found significant differences in all comparisons except for the grip strength of the non-dominant hand. Feder et al. [33] examined handwriting performance at 6 and 7 years in 48 preterm children compared with 69 term children. The children were assessed using the Children’s Handwriting-Manuscript Assessment Tool and various sensorimotor measures and the preterm children had significantly worse scores in legibility and writing speed (*p* < 0.01). Both studies show different results from our research, indicating significant differences between preterm and term children, although it is true that, although they measure grip strength and handwriting performance, we could not compare their results with the “grasping” subscale of the PDMS-II. In this section, there are only six items designed for children older than 36 months, so there is less margin for difference than in the other sections. These items include buttoning and unbuttoning buttons, which the vast majority of children in the study performed successfully, as well as pencil grip when drawing.

In the literature, there are some studies that not only speak of the differences between preterm and term children of preschool and school age, but also name or classify them as MND- or DCD-type disorders, the latter being the most frequent when neurological signs of cerebral palsy are excluded [34]. Arnaud et al. [15] studied the prevalence of MNDs in 1662 very preterm infants, finding an incidence of 44.4%; 31.3% in 245 moderately preterm infants; and 22.6% in 332 term infants. Potharst et al. [16] found statistically significant differences between 104 preterm and 95 term infants with respect to the incidence of MNDs (*p* < 0.01). Broström et al. [14] compared 80 extremely preterm and 90 term infants and found a statistically significant difference (*p* < 0.01) regarding the incidence of MNDs. On the other hand, a review by Edward et al. [17] concluded that DCDs are significantly more prevalent in the preterm and low-birth-weight population compared to term infants. In our research, we did not include the study of these variables, although the results obtained suggest that the children assessed could be great candidates for presenting these alterations. 

On the other hand, Lin et al. [35] assessed the general development of premature children using the Bayley scale of child development at 2 and 5 years of age, and observed unfavorable changes in neurodevelopment from 2 to 5 years of age; more than 70% of children who at 2 years of age had normal or borderline development were borderline at 5 years of age. Gil-Madrona et al. [36] studied five-year-old children born preterm and full term. They observed that prematurity may have influenced some areas of psychomotor development, such as body image, motor dissociation, visual–motor coordination, and social and emotional aspects. These authors advocated for early intervention to try to minimize the consequences of prematurity. Although, in our study, we did not find statistically significant differences between those who attended an early care program and those who did not, due to the fact that the sample was very small and heterogeneous, we agree with these authors and consider it essential to follow up these children in order to detect any type of alteration in time and act as early as possible through early care programs.

Most of the studies found also excluded children with possible neurological damage or pathology associated with prematurity from study; most of them only included very preterm (born from 28 to 32 weeks of gestation) [12], extremely preterm (born below 28 weeks of gestation) [37] or low-birth-weight (<1500 g) [9] children. In our sample, we found no significant correlation between week of gestation at birth and birth weight with respect to the scale scores, although there were no extremely preterm infants: 40% were very preterm and 60% were moderately and late preterm. Tanis et al. [38] analyzed the differences between 28 preterm children with low weight for their gestational age and 28 preterm children with adequate weight for their gestational age; the differences found between both groups were significant in relation to motor skills (*p* = 0.048) and fine motor skills (*p* = 0.021), in contrast to our research, which did not find any correlation between the scale scores and the children’s weight at birth.

The present study has methodological limitations; among them, the heterogeneity of the group and the environment of the participating children were not taken into account. The sample size was limited, and this means that these findings cannot be generalized to the population of premature children in general. Furthermore, more sociodemographic details that could offer deeper insights into the study’s applicability and generalizability should have been collected. On the other hand, the assessment scale used covered only the motor area. It would be interesting to carry out a global developmental assessment in order to better detail the differences found and uncover the real impact of prematurity. 

## 5. Conclusions

The present study allowed us to determine the differences in gross and fine motor development between preterm and full-term children using the PDMS-II scale. Despite the small sample size and the fact that all the children passed the scale and were within the percentiles corresponding to their ages, the results obtained show that at preschool age (3 to 6 years), statistically significant differences continue to be observed with respect to the motor development of these children. 

Likewise, no correlation was found between motor development at preschool age and birth weight and gestational age.

## Figures and Tables

**Table 1:** Descriptive characteristics.

	All	Preterm	At Term
Age in months, mean (SD)	47.93 ± 9.89	49.13 ± 9.49	46.73 ± 10.47
Sex, n (%)	n = 30	n = 15	n = 15
Male	16 (53%)	7 (46.6%)	9 (60%)
Female	14 (47%)	8 (53.33%)	6 (40%)
Gestational week, mean (SD)	32.93 (1.86)		
Birth weight in grams, mean (SD)	1979.6 (549.5)		

SD, standard deviation.

**Table 2:** Comparison of the capacities in the PDMS-II scale between both groups.

Standard Score of the Subscales	Preterm	At Term	*p*-Value
Static balance, median (IQR)	7.00 (2.00)	10.00 (3.00)	0.001 *
Locomotion, median (IQR)	8.00 (2.00)	9.00 (2.00)	0.001 *
Manipulation, median (IQR)	8.00 (2.00)	11.00 (1.00)	<0.01 *
Grasping, median (IQR)	11.00 (3.0)	11.00 (4.00)	0.257
Visual–motor integration, median (IQR)	9.00 (2.00)	11.00 (2.00)	0.011 *
GMQ, median (IQR)	85.00 (10.00)	102.00 (15.00)	<0.01 *
FMQ, median (IQR)	100.00 (12.00)	106.00 (15.00)	0.021 *
TMQ, median (IQR)	90.00 (7.00)	103.00 (11.00)	<0.01 *

GMQ, gross motor quotient; FMQ, fine motor quotient; TMQ, total motor quotient; IQR, interquartile range. * *p*-value < 0.05 using the Mann–Whitney U test for independent samples.

**Table 3:** Comparison of scores on the PDMS-II scale between the group of premature infants who had received early attention and the group who had not received early attention.

Standard Score of the Subscales	Early Care Program Group	No Early Care Program Group	*p*-Value
Static balance, median (IQR)	7.00 (2.50)	7.00 (2.00)	0.864
Locomotion, median (IQR)	8.00 (1.50)	8.00 (2.00)	0.864
Manipulation, median (IQR)	7.50 (2.00)	8.00 (1.50)	0.459
Grasping, median (IQR)	10.00 (3.50)	11.00 (3.50)	0.301
Visual–motor integration, median (IQR)	10.00 (2.50)	9.00 (1.50)	0.077
GMQ, median (IQR)	84.00 (8.50)	85.00 (10.00)	0.401
FMQ, median (IQR)	101.50 (13.50)	97.00 (10.50)	0.476
TMQ, median (IQR)	89.50 (7.50)	90.00 (9.00)	0.953

GMQ, gross motor quotient; FMQ, fine motor quotient; TMQ, total motor quotient; IQR, interquartile range.

**Table 4:** Correlation between the different subscales and ratios and the gestational week and birth weight.

	Gestational Week		Birth weight	
	r	*p*-Value	r	*p*-Value
Static balance	−0.061	0.829	−0.005	0.987
Locomotion	−0.011	0.968	−0.086	0.759
Manipulation	0.388	0.153	0.362	0.185
Grasping	−0.370	0.175	−0.195	0.485
Visual–motor integration	−0.123	0.661	−0.399	0.140
GMQ	0.216	0.440	0.192	0.493
FMQ	−0.396	0.144	−0.455	0.088
TMQ	−0.121	0.668	−0.116	0.680

GMQ, gross motor quotient; FMQ, fine motor quotient; TMQ, total motor quotient.

## Data Availability

The data presented in this study are available upon request from the corresponding author. The data is not publicly available because it is data from children.

## Abbreviations

DCD	developmental coordination disorders
FMQ	fine motor quotient
GMQ	gross motor quotient
MABC	Movement Assessment Battery for Children scale
MND	minor neurological dysfunctions
PDMS-II	Peabody Developmental Motor Scales 2
TMQ	total motor quotient
